# Effectiveness of Grief Camps in Supporting Bereaved Individuals: A Systematic Review

**DOI:** 10.1177/00302228251326146

**Published:** 2025-03-12

**Authors:** Jingyi Chi, Karolina Krysinska, Karl Andriessen

**Affiliations:** 1 Centre for Mental Health and Community Wellbeing, Melbourne School of Population and Global Health, 2281 The University of Melbourne, Melbourne, VIC, Australia

**Keywords:** grief camp, intervention, support, grief, bereavement, mental health, psychosocial, systematic review

## Abstract

Experiencing the death of a loved one is a stressful and disruptive event that can have short-term and long-term detrimental effects on the grief, mental health, and social functioning of the bereaved individuals. Grief camps represent a relatively novel form of support. However, little is known about their effectiveness. The systematic review (PROSPERO: #CRD42024547094) adhered to the PRISMA guidelines and aimed to investigate the effectiveness of grief camps on the grief and psychosocial functioning of participants. Searches in six databases (Medline, Scopus, Web of Science, PsycINFO, Embase, CINAHL) identified 31 relevant studies published between 1991 and 2022. Narrative synthesis of the study findings indicated a positive impact of grief camps on the grief and psychosocial functioning of bereaved individuals, and participants’ feedback indicated that grief camps are an acceptable intervention. Further research in various locations and across different age groups may broaden our understanding of the effects of grief camps.

## Introduction

Bereavement is defined as the experience of losing to death a person to whom one is attached, including parents, spouses, or other loved ones (Hansson & Stroebe, 2003; Stroebe et al., 2007). In most cases, individuals eventually come to terms with the bereavement and continue with their lives carrying the loss of a significant person. However, some bereaved people struggle to recover and the impact of grief can manifest in both psychological and physical ways (Stroebe et al., 2017). Research indicates that bereavement is associated with an increased risk of mental health problems and suicidal behavior (Stroebe et al., 2007; Thimm et al., 2020).

Various grief interventions have been developed to help bereaved individuals improve their physical and psychological health, and to address social isolation (Asgari et al., 2023). Interventions include, but are not limited to, peer support groups, pharmacological therapy, and psychological counseling. Grief camp is a term used to describe a relatively novel form of intervention, consisting of a structured, therapeutic program designed to support bereaved individuals and families (American Camp Association, 2018; Clute & Kobayashi, 2013; National Centre for Childhood Grief, n.d.). Grief camps typically combine traditional camp activities (such as outdoor games, arts and crafts, and team-building exercises) with grief interventions like peer groups, one-on-one counseling, and activities focused on processing grief (American Camp Association, 2018; Clute & Kobayashi, 2013). Grief camps can promote active communication with family, friends, and the community through group interventions (Creed et al., 2001). Bereaved individuals can also receive personalised and multidimensional support through counselling sessions and grief-processing programmes within the camp (Clute & Kobayashi, 2013). Expected outcomes of grief camps include deeper impacts on participants, such as equipping them with coping skills for handling grief, enabling them to manage other challenging emotions and to maintain healthy living (Bachman, 2013). As the literature on grief camps is expanding, synthesis of current evidence is needed to investigate the potential effectiveness of grief camps on the grief and psychosocial functioning of participants.

To address this gap, this review has the following research question: what is the reported impact of grief camps on the grief and psychosocial functioning of participants? As such, this review synthesizes the reported outcomes of grief camps, including provision of meaningful support to bereaved participants, and it may serve as a reference for professionals and organizations involved in providing grief support. It may inform further research and enhance intervention measures and camp activities, ultimately improving the quality of services offered to bereaved individuals.

## Method

This systematic review followed the PRISMA guidelines (Page et al., 2021), and was prospectively registered with PROSPERO, number CRD42024547094, May 25, 2024. The search string was built using the concepts of grief and grief camps. It combined terms such as “grief” “bereave” “mourn” and their derivatives and related terms with “retreat” “residential treatment” “grief camp” “camp” and their derivatives and related terms. Databases used included Medline, Scopus, Web of Science, PsycINFO, Embase, and CINAHL.

The Medline search string combined search words and MeSH was as follows: (Grief/ or grie*.mp. OR Bereavement/ or bereave*.mp. OR mourn*.mp.) AND (retreat*.mp. OR residential. mp. or Residential Treatment/ OR grief camp*.mp. OR camp. mp. OR camps. mp. OR camping. mp. or Camping/). The Scopus search string was as follows: TITLE-ABS-KEY ( grie* OR mourn* OR bereave* ) AND ( TITLE-ABS-KEY ( retreat* OR camping OR residential ) OR TITLE-ABS-KEY ( “grief* camp*” ) OR TITLE-ABS-KEY ( “residential treatment” ) ). The Web of science search string was as follows: ALL=(grie* OR mourn* OR bereave*) AND (ALL=(retreat* OR camping OR residential) OR ALL=(“grief* camp*“) OR ALL=(“residential treatment”)) (exact search). The CINAHL search string was as follows: (retreat* OR camping OR residential OR grief* camp* OR residential treatment ) AND ( mourn* OR grie* OR bereave* ). The search string was slightly adjusted for different database formats, while the search strategy remained consistent.

The search was conducted in June 2024 and was limited to English publications in peer-reviewed journals, without restrictions on publication year or location. Three researchers (JC, KK, KA) used Covidence to screen the search results. The screening process occurred in two steps. First, JC removed the duplicates and the three researchers screened the references based on title and abstract. Next, the three researchers screened the potentially eligible full texts against the inclusion and exclusion criteria. Discrepancies were resolved through discussion. The references of the retrieved papers and existing reviews were manually searched to identify additional studies. Figure 1 presents the search and selection process.

**Figure 1.**
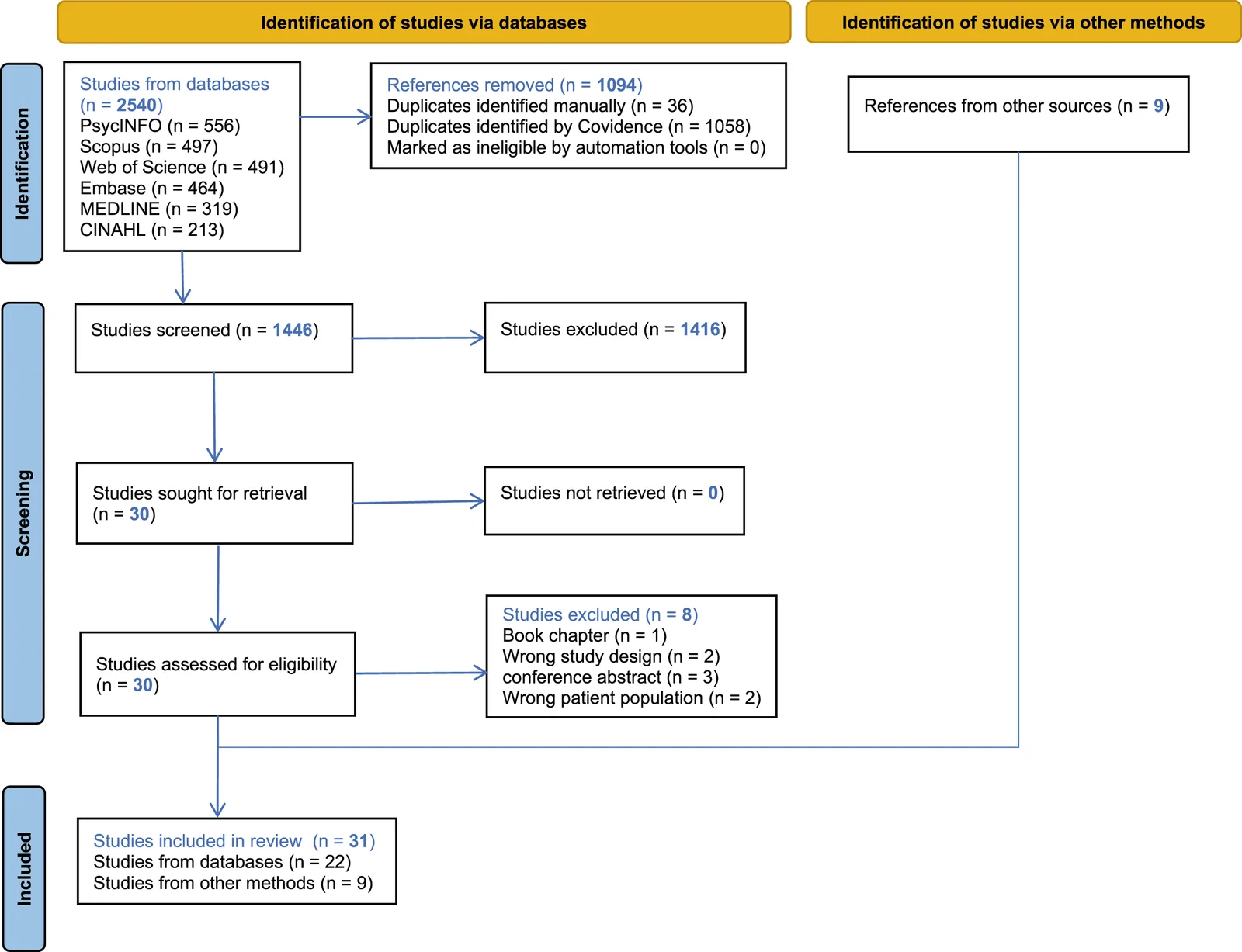
PRISMA Flow diagram.

### Inclusion and Exclusion Criteria

Studies were included if the study: (1) population consisted of bereaved individuals participating in a grief camp; (2) provided empirical data on grief, mental health and/or grief camp-related outcomes; (3) did or did not include a comparator; (4) was published as a full-text paper in a peer-reviewed journal in English. Studies were excluded if the study: (1) did not involve bereaved individuals; (2) did not involve grief camp interventions; (3) did not provide data on bereaved individuals after attending grief camps; (4) used other methods such as case studies or literature review; (5) was not published as a full-text peer-reviewed paper in English.

### Data Extraction

The three researchers collaboratively extracted the following data from the included studies: author (year)/location, sample size for each group, age (mean, standard deviation, range), gender distribution (male/female/other), time since bereavement, relationship to the deceased, type of loss, setting, characteristics of the camp/intervention, duration, outcomes/measures/timepoints, and main results.

### Data Synthesis

We anticipated substantial heterogeneity regarding variables, such as age and gender distribution, time since bereavement, relationship to the deceased, and characteristics of the interventions, which precluded pooling the statistical data. As eligible studies report both quantitative and qualitative data, we adopted a narrative synthesis approach which is recommended in case of heterogeneity across studies (Popay et al., 2006).

### Quality Assessment

All included studies were assessed using the Mixed Methods Appraisal Tool (MMAT) Version 2018 (Hong et al., 2018). The MMAT is an evaluation tool designed for systematic mixed studies reviews, specifically addressing qualitative research, randomized controlled trials, non-randomized studies, quantitative descriptive studies, and mixed methods studies (Hong et al., 2018). It includes the Checklist and the Explanation of the criteria. The answers to the MMAT questions are categorized as Yes, No, and Cannot tell (Hong et al., 2018). Each study must answer two initial screening questions, S1 and S2, which determine whether there is a clear research question and whether the collected data can address the question (Hong et al., 2018). If either S1 or S2 is assessed as No or Cannot tell, further evaluation is unnecessary (Hong et al., 2018).

In the subsequent assessment, different types of studies are evaluated based on five specific questions (Hong et al., 2018). The questions 1.1 to 1.5 are for qualitative studies, the questions 2.1 to 2.5 are for quantitative randomized controlled trials, the questions 3.1 to 3.5 are for quantitative non-randomized studies, the questions 4.1 to 4.5 are for quantitative descriptive studies and the questions 5.1 to 5.5 are for mixed methods studies.

According to the MMAT (2018), the results of the evaluation can be expressed as an asterisk (*) or a percentage (%) based on the answers. For example, one “Yes” out of five questions would equate to 1 * or 20% quality criteria met, and two “Yes” would equate to 2 ** or 40% quality criteria met, three “Yes” would equate to 3 *** or 60% quality criteria met, four “Yes” would equate to 4 **** or 80% quality criteria met, five “Yes” would equate to 5 ***** or 100% quality criteria met.

Since the MMAT (2018) does not provide a clear classification of low, medium, and high quality, this review categorises the quality of studies based on the percentage of “Yes” answers to the five questions (excluding the two initial screening questions, S1 and S2): 100–75% is classified as high quality, 74–50% as medium quality, and 49–0% as low quality. The flexibility of the MMAT lies in its ability to provide specific questions for different types of studies, allowing for a concise and clear comprehensive assessment of articles in a systematic review.

## Results

### Study Characteristics

The systematic search identified 31 relevant studies, with publication years ranging from 1991 to 2022. There were 13 quantitative studies (Table 1), nine qualitative studies (Table 2), and nine mixed-methods studies (Table 3). The studies have been conducted in five countries: 21 studies in the USA, three in Australia, three in Botswana, three in Ireland, and one study in Sweden.

**Table 1.** Summary of Quantitative Studies.

Author (year)/Location	Sample size for each group	Age (M, SD, range)	Gender distribution (Male/Female/Other)	Time since bereavement	Relationship to the deceased	Type of loss	Setting	Characteristics of the camp/ Intervention	Duration	Outcomes/Measures/Timepoints	Main results
Bachman (2013)/USA	First Year: N = 48 Average for Following 3 Years: N = 65	Range 7-17 years	Not reported	Not reported	Children	Various types of deaths	Camp Healing Hearts, A for-profit hospice, Camp Horizon, Facilitated by volunteers from Hospice Care of Kansas and the community	Sustainable, Community-supported, Guidance of the ACE Project/ Arts and crafts, Structured games, Team building exercises, Challenge courses, Whole group sessions, Conversations with peers and camp staff	Weekend	An evaluation tool to measure the impact of the camp on the campers was developed. Follow-up evaluations were done with volunteers and staff. A follow-up with campers and families were done in 30 days, 6 months, and again at 1 year.	Responses suggested campers were able to integrate back into school with decreased stress, and an improved ability to verbalize grief, share their feelings, and trust others. All reported the camp had been helpful and, importantly, fun. Ninety-eight percent of campers reported that camp was a place they felt they were not alone and that they were just normal kids.
Blakley et al. (2018)/USA	N = 8	Not reported	Not reported	Not reported	Family members (parents, siblings, children, spouses)	Homicide	Weekend retreat held at a rural retreat center	Weekend retreat/ Restorative retelling (RR) therapy, Creative arts, Ceremonial grounding exercises	3 days	Psychological outcomes, Overall mental health Timepoints: post-retreat and at six-week follow-up	The condensed RR model worked. Participants reported that the retreat enhanced emotional processing.
Bimbaum (1991)/USA	Children: N = 26 Adults: N = 13 Adolescents: N = 3	Children: Age Range: 2-16 years Adolescents: Age Range: 15-20 years	Not reported	Not reported	Parent loss: N = 17 Child loss: N = 5 Sibling loss: N = 8 Spouse loss: N = 4 Grandparent/Uncle loss: N = 7 Both parents: N = 1	Not reported	A weekend camp in the mountains	Family interaction and peer support, community-building and grief work sessions/ Scavenger hunts, Bird-walks, Aerobic dance, Canoeing, Art, Swimming, Obstacle courses, Hikes, Storytelling, Square-dancing, Campfires	2 days	Likert-scale Timepoints: two weeks after the weekend	Generally positive impact on both children and parents. Increased confidence and sense of mastery through activities. Enhanced social support and movement in the grief process. The evaluations were enthusiastically positive, with almost all elements of the program receiving excellent ratings. Some negative reactions to intense activities like the wish-boat, but these were rare.
Griffiths et al. (2019)/Australia	N = 12	Mean Age: 8.25 years Standard Deviation: 2.15 Age Range: 5-12 years	Males: N = 4 Females: N = 8	Mean time since bereavement was 20.88 months (SD = 23.87)	Children	Sudden death, Illness, Motor vehicle crash, Suicide, Heart attack	Lionheart Camp	Bereavement programs targeting childhood grief/ Psycho-education, Coping strategies, Collaborative activities	2 days	Grief symptomatology: The Inventory of Prolonged Grief for Children (IPG-C) Behavioral problems: The Strengths and Difficulties Questionnaire (SDQ) Parenting and family adjustment: The Parenting and Family Adjustment Scales (PAFAS) Timepoints: Pretest (approximately 2.5 weeks before the camp), Post-test (6–8 weeks after the camp), Follow-up (6 months after the camp)	No significant changes in grief symptomatology. Significant decrease in SDQ peer problems from pretest to post-test. Significant improvements in parental consistency and reduction in coercive parenting from pretest to follow-up and post-test respectively.
Hartwig & Marlow (2021)/USA	N = 30	Mean Age: 10 years Age Range: 7-16 years	Males: N = 12 (40%) Females: N = 18 (60%)	The last 2 years	Immediate family members (e.g., children, siblings, grandchildren)	Not reported	Nonprofit hospice organization	Children’s grief program (CGP)/ Expressive art interventions, Outdoor activities	2 days	Beck Youth Inventories – second edition (BYI-2) at pre- and post-intervention: Beck Depression Inventory (BDI), Beck Anxiety Inventory JOURNAL OF LOSS AND TRAUMA 151 (BAI), Beck Self-Concept Inventory (BSCI)	The findings revealed that the grief camp significantly decreased anxiety and increased self-concept, with no change in depression symptoms. The grief camp merits consideration as an intervention that children’s bereavement programs can use to support grieving youth.
Huss & Ritchie (1999)/USA	Intervention group: N = 9 Control group: N = 8	Age Range: 10 years to 12 years 9 months	Males: N = 9 Females: N = 8	More than 2 years	Children	Suicide, Cancer, Auto accidents	Same suburban, Midwestern, middle-class, middle school	Support group intervention/ Sessions, Establish group rules, Share experiences, Discuss feelings related to the grieving process	6 weeks	Self-esteem: Piers-Harris Self-Concept Scale (PH) Depression: Children’s Depression Inventory (CDI) Behavior: Child Behavior Checklist (CBCL) Self-reported ability to cope with loss: Support Group Rating Scale (SGRS)	No statistically significant changes in self-esteem, depression, behavior, or ability to cope with loss were observed in the participants. A significant increase from pretest to post-test scores on the “I think I know how to cope with loss” was noted. There is evidence from participants’ self-reports and facilitator’s observations that the peer support group helped the participants cope with the death of a parent.
Katisi et al. (2019)/Botswana	N = 650	Mean Age: 13.42 years Standard Deviation: 1.03 Age Range: 11-17years	Males: N = 307 Females: N = 342 One did not disclose	Not reported	Children	AIDS	Balekane EARTH therapeutic intervention in Botswana	Wilderness-based psycho-social strengthening camp/ Group therapy, Art therapy, Individual therapy, Outdoor challenge, Team building activities	2 weeks	The self-report questionnaire was administered at two intervals to cohorts of children at the camps, a day before the intervention program began and 2 weeks later (the last day of camp) Resilience: Child and Youth Resilience Measure (CYRM-28) at pre- and post-intervention Grief: Inventory of Complicated Grief scale at pre- and post-intervention Future Aspirations: Future Aspirations/Peer Leader scale at pre- and post-intervention	Overall resilience scores significantly improved for both male (p=0.001) and female participants (p=0.014), though effect sizes were small (r=0.14 and=0.10, respectively), and only 8% of females and 11% of males demonstrated reliable change. Overall grief scores significantly decreased between the start and end of the intervention for female participants (p=0.004, r=0.12, RC=12.5%) but not for male participants. Participants reporting high grief levels pre-intervention experienced significant reductions in grief post-intervention. Future aspirations showed questionable internal reliability but displayed changes over time for some participants.
Linder et al. (2022)/USA	N = 296	Age Range 11-18 years	Did not report: N = 32 Male: N = 123 Female: N = 141	Not reported	Children	Not reported	In a confidential and secure room allocated by the school	School-based grief group treatment program/ Grief support groups, Curriculum	8 weeks	Social-Emotional Functioning and Validity: The Strengths and Difficulties Questionnaire (SDQ) Emotional Dysregulation: The purpose of the Dysregulation of Emotions Scale (DES) Grief Symptomologyand Reliability: Items for the Grief Symptomology Screener (GSS) Timepoints: Pretest and post-test	No significant difference in pretest scores on the grief symptomology, dysregulation scale, and strengths and difficulties questionnaire for those who completed the group/post-assessment when compared to those who did not complete the group/post-assessment. The result indicated that the mean school-based social support at pre-group (M = 2.15, SD = 1.08) was significantly greater than the mean school-based social support (M = 2.32, SD = 1.1) post-group (p = 0.013). There was a reduction in the frequency of dysregulation, how frequently the students felt angry, anxious, or sad.
McClatchey et al. (2008)/USA	Camp A: N = 46 Camp B: N = 54	Range: 6-16 years	Camp A: Males: N = 25 (54.3%) Females: N = 21 (45.7%) Camp B: Males: N = 23 (57.4%) Females: N = 31 (42.6%)	1-48 months	Children	Expected: Camp A: 17 (37.0%) Camp B: 23 (42.6%) Sudden or violent: Camp A: 29 (63.0%) Camp B: 31 (57.4%)	Weekend bereavement camps in a metropolitan area	Short-term camp-based intervention program/ Trauma-focused grief interventions, Group counseling sessions, Memorial services, Memory art activities	Weekend	Extended Grief Inventory (EGI), UCLA PTSD Index Timepoints: T1 (pre-intervention), T2 (post-intervention), T3 (2 weeks post-intervention)	Children had significantly lower EGI scores after attending Camp A compared with children in the comparison group (camp = 8.94; p = 0.01). The symptoms remained stable without treatment but decreased after treatment. The results suggested that the odds of continuing to experience severe PTSD symptoms were 4.45 higher for children who did not attend the camp compared with those who did (χ2 (1) = 5.23, p = 0.02). Similarly, the odds of experiencing severe grief (as measured by EGI at T2) were 3.58 times greater for children who did not attend the camp (χ2 (1) = 5.08, p = 0.02). Significant decrease in PTSD scores for Camp B participants across all three sub-scales (Reexperiencing, Avoidance, and Arousal).
McClatchey & Raven (2017)/USA	Treatment Group: N = 52 Comparison Group: N = 53	Mean Age: 10.72 years Standard Deviation: 2.85 Age Range: 6-17 Years	Treatment Group: Male: N = 31 (60%) Female: N = 21 (40%) Comparison Group: Male: N = 35 (66%) Female: N = 18 (34%)	Ranged from one month to 57 months (SD=11.30)	Children, grandchildren or sibling	Sudden: 70% Expected: 30%	Bereavement camp	Trauma-informed care/ Recreational camp related activities, Grief-related activities, Counseling sessions based on trauma-focused grief interventions	Weekend	Posttraumatic Growth:The Revised Posttraumatic Growth Inventory for Children (PTGI-C-R) Posttraumatic Stress Disorder Symptoms: The fifth section of the University of California at Los Angeles (UCLA) PTSD Reaction Index for DSM-5 Timepoints: Pretest and post-test (4 weeks after the camp)	The children who attended camp had statistically significantly higher PTG scores than the children in the comparison group at post-test (p=0.04). The children who attended camp had lower total PTSD scores than the children in the comparison group. However, the difference was not statistically significant (p=0.34). There was a statistically significant decrease in PTSD symptoms among males in the treatment group (p<0.04).
McClatchey (2018)/USA	Camp 1: N = 25 Camp 2: N = 30 Camp 3: N = 30	Mean Age: 11.3 years Standard Deviation: 2.8 Age Range: 6-18 years	Males: N = 32 (49.2%) Females: N = 33 (50.8%)	Mean: 12.4 months Standard Deviation: 13.4 Range: 1 month to 48 months	Children: N = 47 (72.4%) Grandchildren: N = 6 (9.2%) Sibling: N = 12 (18.5%)	Expected losses: N = 23 (35.4%) Sudden or unexpected losses: N = 42 (64.6%)	Healing camp for bereaved children held in three different locations in the Southeastern United States	Trauma-informed curriculum/ Counseling sessions, Grief activities, Camp activities	Weekend	PTGI-C (Post-traumatic Growth Inventory for Children) at pretest and post-test (1 month after camp)	Positive impact on post-traumatic growth (PTG) among bereaved children (p < 0.05). Circumstances of death was predictors of PTG, with children who had experienced a sudden or unexpected death showing statistically significantly higher PTG scores (p < 0.01).
Pfeffer et al. (2002)/USA	Intervention Group: N = 39 Non-Intervention Group: N = 36	Age Range: 6-15 Years Intervention Group: Mean Age: 9.6 years Standard Deviation: 2.9 Non-Intervention Group: Mean Age: 11.4 years Standard Deviation: 3.5	Intervention Group: Male: N = 16 (41%) Female: N = 23 (59%) Non-Intervention Group: Male: N = 12 (33.3%) Female: N = 24 (66.7%)	Intervention Group: Mean days: 306 (SD=376) Non-Intervention Group: Mean days: 515 (SD=1008)	Children or sibling	Suicide	Manual-based bereavement group intervention for children who suffered suicide of a parent or sibling	Bereavement group intervention (BGI)/ Group sessions	Not reported	The Schedule for Affective Disorders and Schizophrenia for School-Age Children-Present State (K-SADS-IVR) The Beck Depression Inventory (BDI) The Childhood Posttraumatic Stress Reaction Index (CPTSRI) The Children’s Depression Inventory (CDI) The Revised Children’s Manifest Anxiety Scale (RCMAS) The Social Adjustment Inventory for Children and Adolescents (SAICA) Timepoints: Initial assessment (T1) and follow-up assessment (T2), with time from T1 to T2 ranging from 2.5 to 4.5 months.	Results suggested that age had a curvilinear relation to children’s anxiety (RCMAS) and depression (CDI) scores. Greater reduction in depressive and anxiety symptoms for children who received the intervention compared to those who did not (p ≤ 0.01).
Salloum et al. (2001)/USA	N = 45	Mean Age: 14.3 years Age Range 11-19 years	Male: N = 18 (40%) Female: N = 27 (60%)	Not reported	Children, sibling, cousin or friend	Homicide	Inner-city schools	Group therapy model/ Common symptoms of exposure to violence, Grief education, Healthy coping techniques, Safety, Identifying and sharing feelings, Revenge and anger management, Support systems, Spirituality, Future goals	8-10 weeks	The Child Posttraumatic Stress Reaction Index before and after the intervention	The results of this pilot study suggest that group therapy may be helpful in reducing PTSD symptoms among adolescent survivors of homicide victims. Significant reduction in total Child PTSD Reaction Index scores and two of three symptom clusters at post-test. Gender differences in effectiveness were minimal. Although the lack of a comparison group threatens the study’s validity, the results suggest that group therapy may be helpful in reducing PTSD symptoms for this population.

**Table 2.** Summary of Qualitative Studies.

Author (year)/Location	Sample size for each group	Age (M, SD, range)	Gender distribution (Male/Female/ Other)	Time since bereavement	Relationship to the deceased	Type of loss	Setting	Characteristics of the camp/ Intervention	Duration	Outcomes/Measures/Timepoints	Main results
Gregory et al. (2022)/ USA	N = 10 (six non-bereaved and four bereaved)	Age Range: 7-16 years	Not reported	Not reported	Siblings	Cancer	The non-profit organization partnered with a regional 4-H camp	Peer support camp/ Team-building activities, Creative expression projects, Separate bereaved chat sessions	7 days	The one-to-one semi-structured phone interviews were conducted approximately three to six weeks after the camp	The study found that siblings perceived positive outcomes associated with the peer support camp experience, including social connection, personal growth, and the opportunity to relax and have fun. These outcomes were consistent across age, gender, and bereavement status.
Hanlon et al. (2022)/Ireland	Parents: N = 12 Staff: N = 6	Not reported	Parents: Males: N = 4 Females: N = 8 Staff: Males: N = 2 Females: N = 4	1 to 6 years	Parents	Serious illness	A therapeutic recreation-based bereavement camp	Therapeutic recreation-based bereavement camp/ Large group activities, Mealtimes	4 days/3 nights 1-year period, at six-monthly intervals	Thematic analysis of semi-structured interviews conducted with parents and program staff	The study highlighted positive contributions associated with participation in the program, including coping with bereavement, access to support, and implications for family functioning.
Kramer & Sodickson (2002)/USA	Parents: Not reported Ages 3-5: N = 10 Ages 6-8: N = 8 Ages 9-12: N = 15 Aes 13-17: N = 14	Parents: Not reported Children: Range: 3-17 years	Not reported	Not reported	Parents or siblings	Illness	Walker Creek Ranch, which is a really beautiful outdoor educational facility in northern California	Comprehensive Parent Program, Comprehensive Children’s Program/ Session, Group-building activities, Nondenominational memorial service, Square dance, Nature walk	Weekend	Feedback, Program evaluations and Open-ended questions after retreat	The family retreat offered a place to openly mourn the death. Participants’ grief was validated and struggle was shared by others who truly understood the journey. Parents’ comments shows that it was a valuable support system. Comments from children and teens shows that they liked the Retreat and it was fun.
McClatchey & Wimmer (2012a)/USA	Children: N = 16 Parents: N = 11	Children: Age Range: 8-18 years	Children: Males: N = 11 Females: N = 5	Within the past two years	Children or mate	Not reported	Residential weekend grief camp​	Included a parallel camp for parents and was based on trauma-focused grief camp/ Counseling groups, Memory art work, A memorial service, A balloon release, Traditional camp activities	48 hours	During a two-month period, the semi-structured interviews focused on themes such as sadness, anger, being set apart, worries, trauma, and contemplation of suicide	The children and adolescents get a chance to acknowledge through rituals, counseling sessions, and other activities that loss has occurred. Interventions open avenues for campers to express feelings surrounding the loss as well as provide the campers with tools to deal with their feelings and join with other children who have had similar experiences. This study was not an evaluation of camp and does not claim that specific camp interventions changed the Children’s grief reactions. Other variables, such as family support and passage of time, may also have influenced these changes. Interventions influenced the campers’ sadness, anger, being set apart, worries, trauma, and contemplation of suicide.
McClatchey & Wimmer (2012b)/USA	Children: N = 19 Parents: N = 13	Children Age Range: 8-18 years	Not reported	3 to 9 months	13 children lost a father, 4 lost a mother, 2 lost grandparents	Car accidents, Cancer, Suicide, Heart attack, Accidental drug mixing, ALS, Boating accident	Camp MAGIK	Therapeutic interventions/ Counseling sessions, Memorial service, Balloon release, Journaling, Traditional camp activities	2 days	Semi-structured interviews	The study identified therapeutic interventions and traditional camp activities as significant healing components.
Ross et al. (2019)/Australia	N = 15	Age Range: 31-73 years	Males: N = 3 Females: N = 12	Ranged from 1 year and 3 months to 5 years	Spouse, partner, or family member	Suicide	Focus groups	Focus groups discussing support needs, Interpretive phenomenological analysis (IPA)/ Changing support needs, Difficulties navigating services, Experiences of stigma and social isolation, Connecting with others	Each focus group lasted approximately 90 minutes	Interviews	The study identified key themes regarding the experiences and needs of the suicide-bereaved, emphasizing the importance of tailored support services.
Salinas (2021)/USA	N = 5	Mean Age: 9.8 years Age Range: 7-12 years	Females: N = 5	Within four years	Children or grandchildren	Cancer, Heart failure, Homicide, Terminal Illness	Bereavement Camp	A supportive and therapeutic camp/ Play and Child Development, Sandtray	3 days	Interviews were held approximately three months after the camp ended, focusing on children experiences and coping mechanisms	Children were able to make connections with other participants and memorialize their loved ones. Further, children remembered coping skills they learned at bereavement camp and were able to utilize them at home. Participants enjoyed their experience at bereavement camp. Participants expressed how much they valued the time they had for free play and the trip to the children’s museum. The literature denotes a lack in communication between professional counselors and camp staff in addressing effective grief interventions.
Silvén Hagström, A. (2021)/Sweden	Children: N = 11 Parents: N = 11	Children: Mean Age: 9 years Age Range: 6-13 years	Children: Males: N = 5 Females: N = 6 Parents: Males: N = 2 Females: N = 9	Between 6 months and 5 years, with an average of about 1.5 years	Children or parents	Suicide	Grief support camp organized by the non-profit organization Children’s Rights in Society	Grief support camp/ Parallel group meetings for parents and children, Combined with grief-oriented family exercises and playful activities, Psycho-educational activities	Friday to Sunday, 4 months apart	Narrative structured interviews conducted 18 months after the program ended	Significant positive impact on participants' grieving processes and life. Encounters with other suicide-bereaved led to support exchange and normalization, reducing perceived suicide stigma. Constructing destigmatizing understandings of suicide relieved self-blame and shame. Better-informed position in grief, increased manageability, enhanced family communication. Parents reported improved ability to support their children and a more hopeful outlook on life.
Thamuku & Daniel (2013)/Botswana	Children: Initially 44 orphaned children, later narrowed down to 10 representative participants N = 10 Social Workers: N = 8	Children:Range 13-15 years	Not reported	Not reported	Children	AIDS	EARTH therapeutic retreat in Botswana	EARTH therapy program/ Group counseling, Role plays, Art therapy, Individual therapy, Concluding group therapy, Lecture and discussion based on case studies, Outdoor activities	A wilderness-based therapeutic retreat lasting 16 days and a follow-up support program for 3 years	Children: Semi-structured questionnaires and semi-structured interviews were collected in five rounds Social Workers: Self-administered questionnaires with open-ended questions	Children revealed issues linking experiences of abuse, discrimination, or marginalization with thoughts of late parents and the pain of loss. Children shared experiences of death and reactions to the incidence of death, reflecting on their life stories in relation to their late parents. Children found it difficult to release painful emotions but persisted in sharing with the group, understanding that it would help them cope with their grief.

**Table 3.** Summary of Mixed-Methods Studies.

Author (year)/Location	Sample size for each group	Age (M, SD, range)	Gender distribution (Male/Female/Other)	Time since bereavement	Relationship to the deceased	Type of loss	Setting	Characteristics of the camp/Intervention	Duration	Outcomes/Measures/Timepoints	Main results
Braiden et al. (2009)/Ireland	Adults: N = 7 Children: N = 8	Mean Age: 10 years Age Range: 5-16 years	Adults: Males: N = 2 Females: N = 5 Children: Males: N = 2 Females: N = 6	At least 6 months prior	First-degree relative/ step-relative	Suicide	Residential program organized by Barnardo’s Child Bereavement Service	Residential grief camp/Common Responses to Grief and Trauma, Development and Grief, Emotional First Aid Kit and Support Networks, My Story Strip, Jenga Game, Identifying Feelings and Emotions	Not reported	Qualitative Methods: Interviews with children, parents, and staff Quantitative Methods: Semi-structured questionnaires Timepoints: pre-intervention and post-intervention	Children reported feeling less alone, more confident, and happier. Parents found the residential beneficial for coping with their grief and facilitating open discussions about their loved ones. Staff felt privileged to be involved and reported positive experiences.
Creed et al. (2001)/USA	N = 19	Range 6-15 years Group1: 6-11 years Group2: 12-15 years	Not reported	Within the last 3 years	Siblings	Cancer or a blood disorder	Children's oncology/hematology centers within a 100-mile radius of the CCCBD	Camp New Horizons, a weekend camp/Reduce feelings of isolation, Assist children with the appropriate expression of their grief, Educate children about the grief process, Facilitate moving forward in their grief process	Weekend	Qualitative Methods: Open-ended questions and suggestions Quantitative Methods: Evaluation forms focusing on various camp activities rated on a Likert scale	Most helpful activities: art, company of peers, memory book, and memories and feelings session. Suggestions: extending the duration and inviting more hospitals to participate. Camp New Horizons has helped to meet many of the educational and support needs of bereaved siblings at the CCCBD and has received high evaluation marks from participants, staff, and parents. Parents and children reported an increase in their communication about the death in their family.
Farber & Sabatino (2007)/USA	2003: N = 50 2004: N = 50	Range 6-10 years (54%) 11-15 years (46%)	Not reported	Not reported	Children (44%) Grandchildren (28%) Sibling (17%) Cousins and close friends or peers (the rest)	Homicide, Illness, Self-inflicted harm, Gun-related suicide, Drug overdose, Car accident	A central communal building and group events and is surrounded with multiple-bed cabins located in a rural community of eastern Maryland	Short-term, cognitively oriented psychotherapy model/Handicraft, Group activities, Therapeutic and typical leisure camp activities	3 days	Qualitative Methods: Parents’ or caregivers’ perceptions of children’s camp experiences in psycho-social functioning was assessed by a structured telephone interview Quantitative Methods: Likert rating scale of Children’s Engagement in Camp Therapeutic Activities Children’s psycho-social functioning in camp was measured by an adapted and self- administered pencil and paper 5-point Likert scale, Behavioral Rating Index for Children (BRIC) Timepoints: During camp, One-month follow-up	Parents rated their children as experiencing more problems at one-month follow-up compared to during camp in 2003. In 2004, these children, on average, were showing significantly and substantially reduced levels of difficulties in their psycho-social functioning (p = 0.000). The positive changes evidenced in parents BRIC ratings were also supported by their qualitative comments and by other data that indicated satisfaction with the camp experience.
Hanlon et al. (2019)/Ireland	Parents: N = 13 Staff: N = 6	Mean Age: 42.3 years Standard Deviation: 8.1 Age Range: 27-57 years	Males: N = 4 Females: N = 9	In the last 3 years Range: 2 to 48 months Mean: 20 months SD = 14.4	Parents	Hematological or oncological conditions, mitochondrial disorders	A therapeutic recreation-based bereavement camp, Barretstown, Co. Kildare, Ireland	Therapeutic recreation-based bereavement camp/Structured play for parents and children, Bereavement components, Family activities	4 days/3 nights 1-year period	Qualitative Methods: Interviews after the final camp and reunion camp Quantitative Methods: Open-ended surveys before and after each camp	Shared experiences help in normalization. Camp provides a nonjudgmental environment to share stories and difficulties. Families benefit from the therapeutic recreation and supportive atmosphere. Staff and parent volunteers play a crucial role in the camp's impact.
Henning et al. (2021)/USA	Family camp survey: N = 657 Parent focus group: N = 15	Not reported	Not reported	Family camp survey: 3 months to 19 years Parent focus group: 6-60 months	Parents	Family camp survey: Any cause Parent focus group: Malignancies	Family bereavement camp	Family camp and parent bereavement focus group/Ropes courses, Remembrance service, Semi-structured discussion	Weekend camp twice a year and focus group once every six weeks	Qualitative Methods: Family Camp Survey: Open-ended questions to capture more detailed feedback and personal experiences Parent Bereavement Focus Groups: A semi-structured discussion format to gather in-depth qualitative feedback on the parents' experiences and the helpful aspects of the intervention Quantitative Methods: Family Camp Survey: Likert scale ratings to quantify participants' understanding of the grief process, comfort in communicating about the child's death, and the helpfulness of specific activities.	Parents identified the need for ongoing support and benefited from organized peer support. The intervention increased parents' knowledge of grief. Parents manage grief better after the intervention.
Nabors et al. (2004)/USA	N = 18	Range: 6-12 years	Males: N = 9 Females: N = 9	Not reported	Children or grandchildren	Not reported	Hospice-sponsored grief camp	Hospice/Art activities, Crafts, Games, Sports	3 days	Qualitative Methods: Semi-structured interviews with children during and after the camp Open-ended questions to assess children's opinions about their camp experience Quantitative Methods: Surveys completed by parents before and after camp At a six-month follow-up, using Likert scales and yes/no questions to measure changes in emotional functioning (sadness, worry) Children's satisfaction with specific camp activities (feelings masks and board game) recorded using 4-point Likert scales	Parents reported high levels of sadness and anxiety in their children related to the loss. No significant changes were noted in levels of sadness or anxiety immediately after camp. Children and parents were positive about the camp experience. The study suggested that grief camps provide a supportive environment for children to process grief with peers having similar experiences.
Patterson et al. (2021)/Australia	N = 109	Mean Age: 16.63 years Standard Deviation: 3.39 Age Range: 12-25 years	Males: N = 35 (32.1%) Females: N = 74 (67.9%)	Not reported	Offspring: 88.1% (n = 96) Sibling: 11.9% (n = 13)	Cancer	The Good Grief (GG) program	Camp-based program designed based on the dual process model (DPM) of coping with bereavement/Therapeutic activities, Group discussions, Recreation/respite activities	3 days/2 nights	Qualitative Methods: Open-ended questions and interviews Quantitative Methods: Acceptability: 11-point discrete visual analogue scales, Helpfulness, satisfaction, grief intensity, meaning-making and unmet needs : Likert scale, Trauma coping abilities: Seven-point numeric rating scale Timepoints: T0 (baseline), T1 (post-program), T2 (3 months post-program)	99% of participants agreed that GG was helpful or very helpful, and 99% reported they were mostly or very satisfied with the program. The psycho-social sessions were the most useful aspects of the program. A statistically significant difference in outcome scores from T0 (baseline) to T2 (3 months post-program), p <0.001. Statistically significant improvements in personal growth-related meaning-making, trauma coping abilities, and reduction in unmet needs were reported from baseline to three months post-program.
Thamuku & Daniel (2012)/Botswana	Children: Initially 44 orphaned children, later narrowed down to 10 representative participants N = 10 Social Workers: N = 8	Children:Range 13-15 years	Not reported	Not reported	Children	AIDS	EARTH therapeutic retreat in Botswana	EARTH therapy program/Art therapy using the workbook “Grieving Someone Special”, Individual therapy, Group sessions	A wilderness-based therapeutic retreat lasting 16 days and a follow-up support program for 3 years	Qualitative Methods: Children: Semi-structured questionnaires and semi-structured interviews were collected in five rounds Social Workers: Self-administered questionnaires with open-ended questions Quantitative Methods: Secondary data in the form of NGO documents, reports and correspondence pertaining to the Ark for Children programme since 2001	Rites of passage and rites of affirmation promote psycho-social well-being in participating children by fostering a sense of self-appreciation as well as a strong sense of belonging to the group. Children expressed relief and a sense of achievement after participating in the therapeutic rites. They also improve children’s capacity to use available resources to meet their needs and enable children to cope with personal adversity and adapt to change. They were able to talk about their grief and find solutions to their problems through group support.
Tuck. et al. (2012)/USA	N = 8	Mean Age: 52.2 years Standard Deviation: 8.8 Age Range: 35-61	Males: N = 1 Females: N = 7	1-28 years (Mean = 10 years)	Majority were parents of the victim (n = 6), one was a sibling, and one was a cousin	Homicidal death	TOZI Healing Retreat held at a local retreat center	TOZI Healing Retreat/Alternative therapies, Spiritual practices	28 hours	Qualitative Methods: Feedback on the acceptability and feasibility of the intervention and suggestions for improvement Quantitative Methods: General Well-Being Scale (GWB) Center for Epidemiological Studies– Depression Scale (CES-D) The Impact of Event Scale–Revised (IES) PTSD Checklist–Civilian Version (PCL-C) The Texas Revised Inventory of Grief (TRIG) Spiritual Well-Being Scale (SWB) Herth Hope Index (HHI) Trait Forgivingness Scale (TFS) Transgression-Related Interpersonal Motivations Inventory (TRIM) Single-Item Forgiveness Scale (SIF) Religious Coping Scale–The Brief RCOPE Mental measures include general well-being, depression, PTSD, the impact of the event, and grief (GWB, CES-D, PCL-C, IES, and TRIG, respectively; a total of 98 items) Spiritual measures are open-ended items of religion and spirituality, spiritual well-being, hope, forgiveness (state and trait), unforgiveness, and religious coping (SWB, HHI, SIF, TFS, TRIM, and Brief RCOPE; a combined total of 69 items in six instruments) Timepoints: Baseline, 28 hours post-intervention, 6 weeks, 12 weeks, and 30 months post-intervention	Although depression scores (CES-D) increased immediately post intervention at 28h, possibly due to recall of memories of the homicide, there was a notable 17% decrease from baseline at 6 weeks. Fluctuations could be related to other stressful events experienced during the time frame of the study. In contrast to the depression scores, there was a steady decline noted in all PTSD scores from the baseline mean of 30.63 to 29.88 at 28h, 27.25 at 6 weeks, 26.29 at 12 weeks, and 26.14 at 30 months. The data indicate that the mean scores for the GWB, SWB, TFS, CES-D, HHI, Positive RCOPE, and SIF showed remarkable increases of 4% to 21% and, with the exception of CES-D, this trend is congruent with the positive evaluative statements made regarding the intervention. There were also decreases from baseline in the mean scores of TRIM, PTSD, and Negative RCOPE representing a 2%, 3%, and 12% decline, respectively. These changes are consistent with the theoretical perspective and the a priori predictions of healing outcomes. Possible inferences drawn are the potential immediate benefit of the intervention and the ability to capture the changes with the measures used. There were notable increases from baseline in GWB (moving from “stress problem” to “low positive” ratings; scores highest at 6 and 12 weeks postintervention), HHI, and TFS. There were increases in the objective measure of Spiritual well-being scores (SWB), most notably in the Religious Well-Being subscale. The forgiveness measure of SIF Index was the most strident change reported by the participants. Decreases in scores were noted in overall TRIM (a measure of unforgiveness) and TRIM Revenge and TRIM Avoidance, CES-D, and PTSD. Higher scores were noted on TRIG at 30 months indicating greater grief resolution. Finally, positive religious coping increased markedly over time and only returning to baseline at 30 months, whereas negative RCOPE declined and remained below the baseline score. The intervention was found to be useful regardless of the time since the death of the family member. Participants reported using alternative therapies or spiritual practices post-retreat. The TOZI Healing Retreat was feasible and acceptable based on qualitative responses.

### Study Quality

As shown in Table 4, there were nine quantitative non-randomised studies, nine qualitative studies, nine mixed-methods studies, three quantitative descriptive studies, and one quantitative randomised controlled trial. All 31 studies answered “Yes” to the two MMAT initial screening questions, allowing for further evaluation (Hong et al., 2018). The overall average percentage of “Yes” answered to the final five assessment questions across all studies was 74%. This indicates that the overall quality of the 31 studies was medium to high. When categorised by study type, the nine quantitative non-randomised studies had an average percentage of 62%, indicating medium quality; the three quantitative descriptive studies had an average of 47%, indicating low quality; the one quantitative randomised controlled trial had an average of 60%, indicating medium quality; the nine qualitative studies had an average of 93%, indicating high quality; and the nine mixed-methods studies had an average of 78%, indicating high quality. The average quality of the qualitative studies was the highest, while the average quality of the quantitative descriptive studies was the lowest.

**Table 4.** Quality Assessment.

Author (Year), Location	S1	S2	Q1	Q2	Q3	Q4	Q5
	Are there clear research questions?	Do the collected data allow to address the research questions?					
Quality and risk of bias assessment of included quantitative studies using MMAT criteria for quantitative nonrandomised studies							
			Are the participants representative of the target population?	Are measurements appropriate regarding both the outcome and intervention (or exposure)?	Are there complete outcome data?	Are the confounders accounted for in the design and analysis?	During the study period, is the intervention administered (or exposure occurred) as intended?
Bachman (2013), USA	Yes	Yes	Can not tell	Yes	Can not tell	No	Yes
Griffiths et al. (2019), Australia	Yes	Yes	No	Yes	No	Yes	Yes
Katisi et al. (2019), Botswana	Yes	Yes	Yes	Yes	Can not tell	No	Can not tell
Linder et al. (2022), USA	Yes	Yes	Yes	Yes	No	Yes	Yes
McClatchey et al. (2008), USA	Yes	Yes	Yes	Yes	Yes	Yes	Yes
McClatchey & Raven (2017), USA	Yes	Yes	No	Yes	No	Yes	Yes
McClatchey (2018), USA	Yes	Yes	No	Yes	No	Yes	Yes
Pfeffer et al. (2002), USA	Yes	Yes	No	Yes	No	Yes	Yes
Salloum et al. (2001), USA	Yes	Yes	Yes	Yes	No	No	Yes
Quality and risk of bias assessment of included quantitative studies using MMAT criteria for quantitative descriptive studies							
			Is the sampling strategy relevant to address the research question?	Is the sample representative of the target population?	Are the measurements appropriate?	Is the risk of nonresponse bias low?	Is the statistical analysis appropriate to answer the research question?
Bimbaum (1991), USA	Yes	Yes	Yes	No	Yes	No	No
Blakley et al. (2018), USA	Yes	Yes	Yes	No	Yes	No	Can not tell
Hartwig & Marlow (2021), USA	Yes	Yes	No	No	Yes	Yes	Yes
Quality and risk of bias assessment of included quantitative studies using MMAT criteria for quantitative randomized controlled trials studies							
			Is randomization appropriately performed?	Are the groups comparable at baseline?	Are there complete outcome data?	Are outcome assessors blinded to the intervention provided?	Did the participants adhere to the assigned intervention?
Huss & Ritchie (1999), USA	Yes	Yes	Yes	No	Yes	No	Yes
Quality and risk of bias assessment of included qualitative studies using MMAT criteria for qualitative studies							
			Is the qualitative approach appropriate to answer the research question?	Are the qualitative data collection methods adequate to address the research question?	Are the findings adequately derived from the data?	Is the interpretation of results sufficiently substantiated by data?	Is there coherence between qualitative data sources, collection, analysis and interpretation?
Gregory et al. (2022), USA	Yes	Yes	Yes	Yes	Yes	Yes	Yes
Hanlon et al. (2022), Ireland	Yes	Yes	Yes	Yes	Yes	Yes	Yes
Kramer & Sodickson (2002), USA	Yes	Yes	Yes	Can not tell	Can not tell	Yes	Yes
McClatchey & Wimmer (2012a), USA	Yes	Yes	Yes	Yes	Yes	Yes	Yes
McClatchey & Wimmer (2012b), USA	Yes	Yes	Yes	Yes	Yes	Yes	Yes
Ross et al. (2019), Australia	Yes	Yes	Yes	Yes	Yes	Yes	Yes
Salinas (2021), USA	Yes	Yes	Yes	Yes	Yes	Yes	Yes
Silvén Hagström (2021), Sweden	Yes	Yes	Yes	Yes	Yes	Yes	Yes
Thamuku & Daniel (2013), Botswana	Yes	Yes	Yes	Yes	Yes	Can not tell	Yes
Quality and risk of bias assessment of included mixed-methods studies using MMAT criteria for mixed-methods studies							
			Is there an adequate rationale for using a mixed methods design to address the research question?	Are the different components of the study effectively integrated to answer the research question?	Are the outputs of the integration of qualitative and quantitative components adequately interpreted?	Are divergences and inconsistencies between quantitative and qualitative results adequately addressed?	Do the different components of the study adhere to the quality criteria of each tradition of the methods involved?
Braiden et al. (2009), Ireland	Yes	Yes	Yes	Yes	Yes	No	Can not tell
Creed et al. (2001), USA	Yes	Yes	Yes	Yes	Yes	Yes	Yes
Farber & Sabatino (2007), USA	Yes	Yes	Yes	Yes	Yes	No	Yes
Hanlon et al. (2019), Ireland	Yes	Yes	Yes	Yes	No	Yes	Yes
Henning et al. (2021), USA	Yes	Yes	Yes	Yes	Yes	Can not tell	Yes
Nabors et al. (2004), USA	Yes	Yes	Can not tell	Yes	Yes	No	Yes
Patterson et al. (2021), Australia	Yes	Yes	Yes	Yes	Yes	No	Yes
Thamuku & Daniel (2012), Botswana	Yes	Yes	Yes	Yes	Yes	Can not tell	Yes
Tuck et al. (2012), USA	Yes	Yes	Yes	Yes	Yes	Can not tell	Yes

Each type of study included different weaknesses. In the quantitative non-randomised studies, although most presented the primary outcomes, eight out of nine did not meet the assessment criterion for complete outcome data. None of the three quantitative descriptive studies met the criterion for sample representativeness due to factors like insufficient sample size or regional limitations. The only quantitative randomised controlled trial did not meet the criteria for baseline comparability between groups and blinding. Two out of nine qualitative studies did not meet the assessment criteria for the appropriateness of data collection methods or the adequacy of data supporting all outcomes. In the mixed-methods studies, seven out of nine did not meet the criterion for consistency between qualitative and quantitative findings.

### Target Population

Most studies focused on children and adolescents, with only a few including adults (Hanlon et al., 2019, 2022; Henning et al., 2021; Patterson et al., 2021; Tuck et al., 2012). The reported ages of the populations ranged from 2 years old (Bimbaum, 1991) to 73 years old (Ross et al., 2019), with the most common age range being 6–12 years. Regarding gender distribution, no studies reported genders other than male and female. In most studies, there were more female participants than male.

Of the 31 studies, 13 did not report the time since bereavement. Among those that did, the shortest reported time was one month (McClatchey, 2018; McClatchey et al., 2008), and the longest was 28 years (Tuck et al., 2012). Most of the target populations in the studies had lost a parent or guardian (e.g., grandparents or uncles), while some had lost a sibling, cousin, friend, child, or spouse.

The types of loss reported in the studies could generally be categorised into two groups: expected losses and sudden/unexpected losses. Expected losses included those due to chronic illness, malignant tumors, AIDS, and cancer, while sudden/unexpected losses included those caused by homicide, suicide, drug overdose, traffic accidents, and heart disease.

### Interventions

All included studies employed grief camp interventions, although there were some differences in the specific activities and schedules. Three of the studies evaluated the EARTH therapeutic intervention in Botswana and its outcomes (Katisi et al., 2019; Thamuku & Daniel, 2012, 2013). The EARTH program mainly targeted orphans who had lost their parents to AIDS, providing them with a two-week grief camp intervention (Katisi et al., 2019). Another five studies focused on individuals who had lost loved ones to suicide, addressing their grief and psychological issues (Braiden et al., 2009; McClatchey & Wimmer, 2012a; Pfeffer et al., 2002; Ross et al., 2019; Silvén Hagström, 2021). Addressing specific features of this type of bereavement, these studies reported targeted interventions addressing experiences of stigma and social isolation (Ross et al., 2019). Three studies targeted individuals who had lost loved ones to homicide (Blakley et al., 2018; Salloum et al., 2001; Tuck et al., 2012). These studies reported specific activities, such as sessions on common symptoms of exposure to violence and on revenge and anger management (Salloum et al., 2001).

Most studies included activities which may be typical of traditional camps, such as team-building activities (working together to complete tasks), arts and crafts (creative expression of emotions), and outdoor activities (such as boating, hiking, and group dancing). Activities specific to grief camps included grief education (identifying feelings and emotions, expressing grief), grief support groups (sharing and discussing experiences and feelings in groups), counselling sessions (individual guidance and support), memorial services (collectively remembering loved ones), and individual therapy.

The reported duration ranged from 28 hours (Tuck et al., 2012) to 10 weeks (Salloum et al., 2001). Most interventions were weekend camps, with a total of 12 studies having a duration of two days. Six others had a duration of three days (Blakley et al., 2018; Farber & Sabatino, 2007; Nabors et al., 2004; Patterson et al., 2021; Salinas, 2021; Silvén Hagström, 2021), three lasted for two weeks (Katisi et al., 2019; Thamuku & Daniel, 2012, 2013), and two others spanned four days (Hanlon et al., 2019, 2022). The duration of the other studies either fell within the aforementioned range or was not reported.

### Grief Outcomes

Several studies have assessed the impact of grief camps as an intervention on participants’ grief. McClatchey et al. (2008) conducted a study on grief camps in the USA using the Extended Grief Inventory (EGI) to evaluate both participants and a control group. The results showed a significant reduction in EGI scores for participants (*p* = .01); individuals who did not attend the camp were 3.58 times more likely to experience severe grief (*p* = .02). This suggested that grief camps have a positive impact on alleviating grief and may help prevent severe grief experiences to some extent. In Botswana, Katisi et al. (2019) used the Inventory of Complicated Grief scale to assess participants before and after a grief camp intervention. Those with higher levels of grief before the intervention saw a more significant reduction in grief after the camp (Katisi et al., 2019). This indicated that grief camps may be more beneficial for individuals with higher initial levels of grief. Additionally, the study showed that female participants experienced a significant reduction in grief scores (*p* = .004, *r* = 0.12), while male participants did not (Katisi et al., 2019), which suggested that grief camps may have a greater effect on reducing grief symptoms in women than in men. However, Gregory et al. (2022) found no differences in the effects of grief camps across age, gender, or bereavement status in their study conducted in the USA. Griffiths et al. (2019) reported no significant change in grief status when using The Inventory of Prolonged Grief for Children (IPG-C) to evaluate participants in Australian grief camps before and after the intervention. Similarly, Linder et al. (2022), using the Grief Symptomology Screener (GSS), found no significant difference in grief symptoms between participants and non-participants in grief camps in the USA. Nabors et al. (2004) also reported that grief camps did not affect participants’ levels of grief. Unlike the assessment scales commonly used in quantitative studies, qualitative research mostly relied on interviews to evaluate bereaved individuals. Interestingly, in all the qualitative and mixed-methods studies included in this review, bereaved individuals responded positively when asked whether they found grief camps effective. Overall, qualitative findings indicated that grief camps had a positive influence on participants’ grief, including experienced improvements in their grief symptoms, grief processes, and daily lives (McClatchey & Wimmer, 2012a; Silvén Hagström, 2021). However, this finding differs from the results of some quantitative studies using assessment scales.

The studies also explored the impact of grief camps on participants’ understanding of grief and their ability to cope. Hanlon et al. (2022) highlighted the positive role that grief camps in Ireland played in helping participants cope with the pain of bereavement. Similarly, Silvén Hagström’ s (2021) study in Sweden demonstrated that grief camps aided participants in better defining and managing their grief. Henning et al. (2021) supported this view, suggesting that the interventions within grief camps increased participants’ awareness of grief, enabling them to handle it more effectively post-intervention. Furthermore, Bachman (2013) conducted pre- and post-intervention evaluations of participants in a two-day grief camp in the USA, revealing that these camps enhanced participants’ ability to express their grief. Kramer and Sodickson (2022) reported that participants were more inclined to share their experiences and inner struggles with others in the camp, compared to individuals in their everyday lives. This may be due to the camp providing more opportunities for relaxation and recreation (Gregory et al., 2022). Additionally, studies indicated that participating in grief camps with others who had similar experiences fostered empathy among participants, and the mutual support between peers helped them process their grief more effectively (Braiden et al., 2009; Nabors et al., 2004). Similar findings were reported by Thamuku and Daniel (2013; 2012), where participants initially struggled with grief, but through team support and shared experiences in the camp, their ability to cope with grief improved. Therapeutic interventions combined with traditional camp activities were highlighted as crucial components for achieving these positive outcomes (McClatchey & Wimmer, 2012b). Ross et al. (2019), in their study on grief camps for those bereaved by suicide, further emphasised the need for tailored support services to meet the specific needs of this group.

### Psychosocial Outcomes

Some studies indicated that participants found the psychosocial sessions to be the most beneficial part of the grief camp intervention, with results showing an improvement in participants’ psychosocial health levels post-intervention (*p* < .001) (Patterson et al., 2021). This view is further supported by the findings of Farber and Sabatino (2007) and Thamuku and Daniel (2012), who reported that grief camps significantly reduced difficulties in psychosocial functioning (*p* = .000), fostering participants’ self-appreciation and sense of belonging, thus aiding them in recovering from the pain of bereavement. More specific psychosocial outcomes include posttraumatic stress disorder (PTSD), anxiety, and depression (Hartwig & Marlow, 2021; Huss & Ritchie, 1999; McClatchey, 2018; McClatchey et al., 2008; McClatchey & Raven, 2017; Pfeffer et al., 2002; Salloum et al., 2001; Tuck et al., 2012).

Many studies have provided evidence for the impact of grief camps on symptoms of PTSD. Tuck et al. (2012) noted that PTSD scores showed a steady decline when evaluated at different intervals: pre-intervention (mean PTSD score 30.63), 28 hours post-intervention (28.88), six weeks post-intervention (27.25), twelve weeks post-intervention (26.29), and thirty months post-intervention (26.14). This indicates that grief camps can effectively alleviate PTSD symptoms in bereaved individuals on a long term. Further evidence comes from McClatchey et al. (2008), who assessed the likelihood of severe PTSD symptoms in participants and non-participants, as well as changes in PTSD levels pre- and post-intervention. The results indicated that non-participants were 4.45 times more likely to experience symptoms than participants (*p* = .02) (McClatchey et al., 2008). Moreover, the PTSD scores of participants significantly decreased after the intervention (McClatchey et al., 2008). This suggests that grief camps not only improve PTSD symptoms but may also help prevent participants from developing severe PTSD responses. Additionally, a grief camp study targeting adolescents bereaved by homicide assessed participants before and after the intervention, found reduced PTSD symptoms after the intervention (Salloum et al., 2001).

Several studies have explored the impact of grief camps on anxiety, depression, self-concept, and self-esteem. Pfeffer et al. (2002), in their study of grief camps for children in the USA, assessed participants and non-participants. Participants experienced lower levels of depression and anxiety post-intervention (*p* ≤ .01) (Pfeffer et al., 2002). Furthermore, there was a curvilinear relationship between age and scores for anxiety and depression in children, indicating that grief camps have a positive impact on anxiety and depression, with age potentially influencing the effectiveness of the intervention. This relationship is not a simple linear one (i.e., where the impact increases with age), rather, the effect fluctuates across different age ranges. According to the relational curve, children aged 7–10 may experience the greatest reduction in anxiety and depression through play-based interventions in grief camps. The impact appears to decrease for children aged 11–13, while adolescents aged 14–16 may not benefit significantly from short-term interventions and may require long-term therapy and post-camp support (Hartwig & Marlow, 2021). Hartwig and Marlow (2021) assessed their participants before and after the intervention, and found a significant reduction in anxiety and an increase in self-concept, although there was no significant change in depression symptoms (Hartwig & Marlow, 2021).

Similarly, some research revealed no change in anxiety levels among participants following a grief camp (Nabors et al., 2004). Huss and Ritchie (1999) noted in their study that there were no statistically significant changes in self-esteem or depression before and after the intervention. In relation to the lack of change in anxiety and depression symptoms before and after grief camps, Tuck et al. (2012) suggested that while there was no change or even an increase in depression scores 28 hours post-intervention, this could be due to the emotional distress triggered by recalling memories of bereavement or other stressful life events during the study period. However, the same study also noted a significant reduction in depression scores by the sixth week after the intervention (a 17% decrease), suggesting that grief camps can have a positive effect on depressive symptoms over time. McClatchey and Wimmer (2012a) also noted that grief camps affected participants’ thoughts and emotions, such as anger, separation, worry, trauma, and suicidal feelings, further indicating the positive psychological impact of these camps on reducing negative emotions.

A few studies reported the reduction of grief camp participants’ stress levels (Bachman, 2013). Tuck et al. (2012) found that participants showed improvements in stress-related issues post-intervention, with these changes becoming more pronounced at six and twelve weeks after the camp, suggesting a potentially lasting effect.

Studies found positive impact of the grief camp intervention on resilience. Katisi et al. (2019), in their study of grief camps in Botswana, assessed participants before and after the intervention, with results showing significant increases in overall resilience scores for both females (*p* = .014) and males (*p* = .001). Braiden et al. (2009) similarly noted that participants in grief camps felt more confident and happier. This suggests that the activities provided in grief camps help participants build resilience, enabling them to better process negative emotions and enhance their self-confidence.

Another positive outcome is the rekindling of hope for the future and life itself. Silvén Hagström (2021) reported that grief camps helped participants regain hope for the future. In their study, Katisi et al. (2019) assessed participants before and after the intervention, with results showing increased aspirations for the future. Although the internal reliability of this result is uncertain, Katisi et al. (2019) aimed to examine the long-term impact of grief camps on participants’ future aspirations. While short-term assessments cannot provide definitive insights into participants’ ambitions for the future, post-camp evaluations did suggest that grief camps instill renewed hope for life and the future in participants.

Other reported psychosocial benefits include increased self-confidence and a sense of control over one’ s life (Bimbaum, 1991), reduced feelings of shame and guilt related to suicide for participants bereaved by suicide, leading to greater understanding and destigmatisation of suicide (Silvén Hagström, 2021), and enhanced mental well-being and forgiveness among participants bereaved by homicide, with significant psychological changes noted in their ability to forgive (Tuck et al., 2012).

### Behavioral and Developmental Outcomes

Some studies suggest that the positive effects of grief camps on participants include social connections, personal growth, receiving support, and improved family functioning in behavioral and developmental aspects (Gregory et al., 2022; Hanlon et al., 2022).

Research from Hanlon et al. (2019) and Silvén Hagström (2021) both found that sharing experiences and interacting with others during the camp helped participants normalise their lives and social relationships. Bereaved parents improved their parenting skills, and families enjoyed a more therapeutic and supportive atmosphere. Additionally, Salinas (2021) and McClatchey and Wimmer (2012a) observed that participating in activities with others who had similar experiences enabled participants to establish stronger connections with others. Findings from Griffiths et al. (2019) also showed a significant reduction in peer problems post-intervention, indicating that grief camps’ peer support groups, or similar group interventions, effectively help participants restore and enhance their communication and social abilities. Grief camps provide a supportive environment for both therapeutic activities and entertainment, helping bereaved families as well. Moreover, participants who improved their social connections at the camp had higher post-intervention scores on the SDQ’ s social support measure (*M* = 2.32, *SD* = 1.1; *p* = .013) compared to pre-intervention scores (*M* = 2.15, *SD* = 1.08) (Linder et al., 2022). Bimbaum’ s (1991) study provided additional evidence that grief camps can strengthen the positive effects of social support, likely because participants became less isolated and more open to receiving care from others.

Bereaved participants, especially children, may have faced discrimination or marginalisation, leading to emotional withdrawal (Thamuku & Daniel, 2013). Blakley et al. (2018), in their study of grief camps in the USA, reported that participants demonstrated improved emotional regulation post-intervention. Linder et al. (2022) supported this view, noting a reduction in the frequency of emotional dysregulation, including feelings of anger, anxiety, or sadness. This suggests that grief camps help participants manage negative emotions, alleviate their impact, and promote emotional stability.

Grief camps help them cope with personal adversity and sudden life changes (Thamuku & Daniel, 2012). Post-intervention behavioral changes indicated that grief camps helped participants return to normal life, aligning with the study’ s qualitative findings (Farber & Sabatino, 2007).

Studies also evaluated posttraumatic growth (PTG). McClatchey and Raven (2017) found that participants’ PTG scores were higher than the control group post-intervention (*p* = .04), although the reduction in PTSD scores in participants compared to the control group was not significant (*p* = .34). This indicates that grief camps can promote posttraumatic growth, irrespective of PTSD symptoms. McClatchey’ s (2018) study also assessed posttraumatic growth in bereaved children, yielding positive results (*p* < .05). The study further suggested that the nature of the death was a predictor of PTG, with participants who experienced a sudden or unexpected death showing a significant increase in PTG scores (*p* < .01) (McClatchey, 2018).

Other studies have shown that participants continue to use trauma/grief coping skills learned in the camp after it has ended, contributing to their long-term ability to handle trauma (Patterson et al., 2021; Salinas, 2021). This suggests that grief camps offer potentially long-term benefits to participants’ trauma coping abilities.

### Participants’ Perspectives on Grief Camps

Many studies, through qualitative methods such as interviews and open-ended questions, examined the views of participants and staff regarding grief camps. Numerous participants across studies have explicitly stated that they felt that grief camps are beneficial and effective (Bimbaum, 1991; Blakley et al., 2018; Creed et al., 2001; Gregory et al., 2022; Hanlon et al., 2022; Hartwig & Ritchie, 1999; Hartwig & Marlow, 2021; Nabors et al., 2004; Patterson et al., 2021). Participants often described camps as enjoyable and a place where they did not feel isolated, allowing them to fully engage in activities (Bachman, 2013; Creed et al., 2001; Kramer & Sodickson, 2002; Nabors et al., 2004; Salinas, 2021). In some studies, as many as 98% or 99% of participants agreed with this view, with the same percentages expressing high satisfaction with the intervention activities (Bachman, 2013; Patterson et al., 2021). Feedback from staff also indicated that they had positive experiences in the camps and gave high ratings to the activities provided (Braiden et al., 2009; Creed et al., 2001). Based on the results of interviews and open-ended responses, peer support groups were considered to be the most impactful aspect of grief camps (Gregory et al., 2022; Huss & Ritchie, 1999). In Salinas’ s (2021) study, child participants particularly enjoyed free play and visits to children’ s museums. Hanlon et al. (2019) found that in an Irish grief camp, both children and parents identified art, peer companionship, memory books, and memory and feelings sessions as the most helpful activities. Patterson et al. (2021) reported that in an Australian grief camp, participants considered the psychosocial programmes to be the most useful intervention.

However, some areas for improvement in grief camp interventions were identified. In Bimbaum’ s (1991) study, the “Wishing Boat” activity received negative feedback for being ‘too intense’. Salinas (2021) found that communication between professional counsellors and camp staff was lacking when it came to helping participants effectively address grief issues. In Henning et al.’ s (2021) research, parents requested ongoing support and organised peer support for families. Creed et al. (2001) suggested extending the two-day grief camp and involving more hospitals in the programme.

## Discussion

The literature on grief camps has been expanding over the last decades. This systematic review aimed to provide a comprehensive synthesis of the effectiveness of grief camps in supporting bereaved individuals and included 31 studies published over 30 years. The demographic differences in the included studies mainly relate to age and type of bereavement.

Studies on children aged 6–12 by McClatchey et al. (2008) and Katisi et al. (2019) found that grief camps improved emotional regulation, social support, and grief symptoms. Among adolescents aged 13–17, Katisi et al. (2019) reported improvements in resilience and future aspirations, while Linder et al. (2022) found no significant changes in grief symptoms post-intervention. For adults, Hanlon et al. (2022) examined parents who had lost a child and found that grief camps enhanced their understanding of grief and improved communication among family members. As different age groups require different types of intervention support, future grief camps could adjust their activities to better meet these varying needs.

The types of bereavement covered in this review include the loss of loved ones due to chronic illnesses such as cancer and AIDS (Katisi et al., 2019), as well as traumatic losses caused by suicide or homicide (Salloum et al., 2001; Silvén Hagström, 2021). Different causes of bereavement may influence the specific support needs of bereaved individuals. For example, those who have lost someone to suicide may experience more severe social stigma and blame, whereas homicide survivors may struggle with anger and thoughts of revenge (Salloum et al., 2001). Therefore, designing specialised interventions for different types of bereavement may enhance the effectiveness of grief camps.

The type of camp included in the studies may also affect intervention outcomes. For example, Patterson et al. (2021) examined the “Good Grief Program”, which provides support for children experiencing all types of bereavement. Other camps are designed for specific bereaved groups, such as Braiden et al. (2009), which focused on suicide survivors and incorporated targeted psychoeducation on suicide stigma. Specialised camps tailored to particular bereavement experiences may offer more effective support.

There are variations in the findings across the included studies. Quantitative research typically employs standardised measurement tools, such as assessment scales, to determine the effectiveness of grief camps based on changes in bereaved individuals’ scores before and after participation. While many quantitative studies, such as McClatchey et al. (2008) using the Extended Grief Inventory (EGI) and Katisi et al. (2019) using the Inventory of Complicated Grief (ICG) scale, indicate the effectiveness of grief camps, other quantitative studies have reported no significant improvement in grief symptoms (Griffiths et al., 2019; Linder et al., 2022). These discrepancies may be attributed to demographic differences in the study samples, variations in measurement tools, or differences in the structure of grief camps.

In contrast, qualitative studies often use interviews, which provide deeper insights into participants’ experiences and offer suggestions for improvement. For example, Silvén Hagström (2021) did not report specific numerical changes but, through narrative structured interviews, demonstrated that bereaved individuals benefited from grief camps in ways such as improving their ability to cope with grief and developing a better understanding of death, ultimately supporting their grief processing and future well-being.

Mixed-methods studies incorporate both quantitative and qualitative findings, offering a more comprehensive evaluation. For instance, while Nabors et al. (2004) found no significant changes in anxiety and grief levels in their quantitative assessment, their qualitative findings indicated that bereaved individuals and their families had positive experiences in the grief camp and valued the supportive environment.

An analysis of the included studies suggests several factors that may be associated with the effectiveness of grief camp interventions on participants grief and psychosocial functioning. First, the activities within grief camps play a crucial role. Structured therapeutic activities tend to have a more significant impact, such as trauma-focused interventions (McClatchey & Raven, 2017), grief-related memory interventions (McClatchey & Wimmer, 2012a), and psycho-education (Griffiths et al., 2019). Creative and experiential activities, such as art therapy and outdoor challenges, have demonstrated positive effects in both qualitative and quantitative studies (Katisi et al., 2019; Thamuku & Daniel, 2013). Additionally, group-based interventions that foster social connections, such as peer support and group collaboration, have been identified as key components of effective grief camps (Huss & Ritchie, 1999; Ross et al., 2019; Silvén Hagström, 2021).

Regarding study designs, quantitative studies using standardised measurements were more likely to report statistically significant positive outcomes for grief camp interventions. Although mixed-methods studies sometimes found no statistically significant changes in quantitative assessments, their qualitative findings consistently highlighted bereaved individuals’ subjective positive experiences following participation in grief camps (Farber & Sabatino, 2007; Patterson et al., 2021).

Grief camps typically consist of group interventions. Krysinska et al. (2024) examined the perceived impact of a group-based grief intervention, and found that the intervention fostered peer support and emotional expression, which can alleviate grief-related symptoms, which is a finding consistent with the results of this review. Review of the literature also highlights the potential benefits of bereavement group interventions; however, the overall evidence of their effectiveness regarding participants’ grief is still weak (Maass et al., 2022). While group interventions can help bereaved individuals feel less isolated and normalize their grief reactions, they may lack a personalised approach (Johannsen et al., 2019).

Most studies in this review have been focused on grief camps for children and adolescents. The literature indicates that grief interventions tend to be more effective for children and adolescents with higher levels of grief and distress (i.e., therapeutic interventions) compared to those with lower levels (i.e., preventive interventions) (Hanauer et al., 2024; Rosner et al., 2010). Some studies in our review also reported positive impacts of grief camps on children’s high level of grief (e.g., Katisi et al., 2019) and PTSD (e.g., McClatchey et al., 2008). Further research is needed to examine the role of grief and distress levels of children participating in grief camps. In line with consensus best-practice recommendations on how best to support bereaved adolescents (Ross et al., 2021), several grief camps in this review also provided support to parents/guardians. This may contribute to the effectiveness of the intervention as it enhances the parents’ knowledge and skills to support their bereaved children (e.g., Pfeffer et al., 2002).

The needs of bereaved individuals may vary, at least partly, due to the type of bereavement, and several grief camps have been designed for particular groups, including people bereaved by suicide (e.g., Silvén Hagström, 2021), or homicide (e.g., Salloum et al., 2001). The metareview of Hofmann et al. (2024) identified several components of suicide bereavement interventions perceived as effective and similar components have been reported in (suicide) grief camp studies included in this review. These included the use of homogenous groups, manualized approaches, involvement of skilled facilitators and peer support, and psychoeducation (e.g., Braiden et al., 20090; Silvén Hagström, 2021). Potentially effective support for people bereaved by homicide also includes features that have been reported in studies of homicide grief camps (e.g., Salloum et al., 2001), such as manualized interventions, restorative retelling, and psychoeducation (Alves-Costa et al., 2021). Though these findings seem promising, further research is needed to examine the effect of grief camp interventions in alleviating the impact of traumatic types of bereavement.

The effectiveness of grief interventions is often associated with the duration of the intervention, with longer-term interventions generally producing better outcomes (Andriessen et al., 2019). Grief camps, as short-term interventions (weekend or three-day camps), can provide participants with emotional support and group interaction, impacting bereaved individuals’ grief and psychosocial well-being. However, the short duration may limit the depth of emotional expression and long-term support, meaning the effects may not be as profound or lasting as those of more structured interventions, such as cognitive-behavioral therapy (Kaiser et al., 2022). Nonetheless, this review found that grief camps help bereaved participants develop coping skills for grief, which may have lasting benefits. The positive effects of teaching bereaved individuals to express grief and develop coping skills has also been noted in the broader grief literature (e.g., Ross et al., 2021; Stroebe et al., 2001). Nonetheless, grief camps often lack long-term follow-up, which indicates a need for more longitudinal studies.

Several grief interventions, such as those based on cognitive-behavioral therapy (CBT), adapt a highly structured approach with clear steps and predetermined goals (Johannsen et al., 2019; Kaiser et al., 2022). In contrast, grief camps usually are less structured interventions, which offer more flexible activities and schedules to cater for the diverse needs of participants. Our findings indicate that combining therapeutic interventions with traditional camp activities is effective in improving grief, psychosocial, and behavioral outcomes for both individuals and families (e.g., McClatchey & Wimmer, 2012b). On one hand, offering interventions in a more flexible format may allow for a more personal approach, as compared to structured interventions, and may allow bereaved individuals to better express themselves and to participate more freely. On the other hand, such approach may affect effectiveness due to the lack of goal orientation and detailed planning, particularly for those in need of special type of support and guidance, such as those experiencing complicated grief (Kaiser et al., 2022). Further research may explore the balance of structured and unstructured activities needed in grief camps relative to the target group.

It has also been noted that grief interventions may involve various approaches, such CBT, complicated grief group therapy (CGGT), and art therapy (Asgari et al., 2023; Johanssen et al., 2019). These approaches mostly focus on unidimensional interventions: CBT addressing cognitive issues, CGGT focusing on alleviating grief, and art therapy concentrating on emotional expression and coping skills. In the 31 studies on grief camps, different interventions were integrated, such as art therapy and counselling. While multidimensional interventions may have positive impacts across different areas, it may also become difficult to untangle the effectiveness of each individual approach.

### Implications for Practice

Several suggestions can be made for the future development of grief camps. First, activities should be tailored to specific age groups and type of bereavement to offer more personalised support. Second, extending the duration of camp activities could provide individuals and families with more time for relaxation and healing. Third, improving communication between camp staff and counsellors can enhance support for participants. Fourth, feedback from previous camps should be considered to refine activities and ensure their relevance to the needs of participants. Finally, creating more opportunities for families to participate in grief camps together can foster a supportive environment that encourages open communication and healing within the family unit.

### Limitations

This systematic review focused on studies on grief camps from peer-reviewed English-language journals. Although this review includes qualitative, quantitative, and mixed-method studies, which provide a comprehensive understanding of the effectiveness of grief camps, differences in measurement tools may lead to variations in results. Qualitative studies focus on gaining a deeper understanding of participants’ experiences but may lack generalizability. Quantitative studies emphasize numerical changes; however, they may be insufficient in analyzing the complexity of grief and intrinsic emotional experiences. While mixed-method approaches integrate both aspects, variations in research methods and data analysis present challenges in synthesizing findings.

Additionally, the overall quality of the studies varied across low, medium, and high levels. Lower-quality studies may have greater bias and should be interpreted with caution. Future research on grief camps should adopt rigorous study designs and data collection methods.

While all studies utilised validated and reliable measures, confounders were not always addressed, as the studies did not control for interventions other than the grief camps. As such, participants’ outcomes might have been influenced by other interventions, such as counselling or general practitioner services, particularly in studies with longer follow-up periods.

Research reported in this review used mostly pre- and post-assessment design and controlled studies involving participants and non-participants, both of which have limitations. These include time bias as results obtained from pre- and post-intervention assessments may not accurately reflect the long-term impact of grief camps. The grief levels and psychological states of participants may change over time after the intervention, making the results appear more significant than the actual effect of the camp itself (Holland & Neimeyer, 2010).

There may be a regional bias in the population and interventions as 21 of the 31 studies were conducted in the USA. Additionally, most of the studies focused on children. There can be a financial bias, as some individuals interested in attending grief camps may be unable to afford costs associated with transportation and other expenses. There may also be selection bias as control group of non-participants may be less inclined to engage in interventions, such as grief camps, compared to the intervention group. Those who choose to attend grief camps may already be more willing to accept therapeutic interventions and may have stronger social support (Currier et al., 2008).

## Conclusions

This systematic review found promising quantitative and qualitative findings, which suggest effectiveness of grief camps for bereaved individuals, particularly in areas of grief, psychosocial functioning, and behavioral development. Feedback from participants indicated that grief camps are an acceptable intervention that can contribute to improving their grief outcomes and psychosocial well-being. The overall quality of the studies was rated as moderate to high, making the results relatively reliable. Nonetheless, the research is skewed towards the USA and children, indicating a need for more studies in other regions and different age groups to better understand the effects of grief camps in a broader context.
